# Efficacy and Safety of Botulinum Toxin Type A in Primary Axillary Hyperhidrosis: A Meta-analysis and Systematic Review

**DOI:** 10.1007/s00266-025-04909-6

**Published:** 2025-06-11

**Authors:** Jing Sun, Shuangyu Chen, Ting Yang, Liping Huang, Jing Tang, Yuebi Feng, Qiuyue Yu, Yu Wang, Yuanyuan Luo, Yao Tang, Lin Gao

**Affiliations:** 1 Department of Dermatology, Pidu District People’s Hospital, The Third Affiliated Hospital of Chengdu Medical College, Chengdu, Sichuan China; 2 Department of Skin Beauty, Chengdu High Tech Milan Baiyu Medical Beauty Hospital, Chengdu, Sichuan China; 3 Department of Plastic Surgery and Cosmetic Dermatology, West China Hospital of Stomatology Sichuan University, Chengdu, Sichuan China; 4 Department of Dermatology, Honghui Hospital Xi’an Jiaotong University, Xi’an, Shaanxi China

**Keywords:** Botulinum toxin A, Botox, Dysport, Primary axillary hyperhidrosis, Randomized controlled trial, Meta-analysis

## Abstract

**Background:**

The clinical efficacy of botulinum toxin type A (BTX-A) injections for the treatment in primary axillary hyperhidrosis is a subject of ongoing debate. This study aims to consolidate and analyze the available evidence regarding the use of BTX-A as a therapeutic intervention for this conclusion.

**Method:**

This study was conducted in accordance with PRISMA guidelines and registered with PROSPERO. We included RCTs on BTX-A injections for PAH, comparing it to placebo or other treatments. A comprehensive literature search was conducted in multiple databases up to May 2024. Out of identified articles, some underwent full-text review and were included in the qualitative and quantitative synthesis. Statistical analyses were done using RevMan5.4, and study quality was assessed with the Cochrane risk of bias tool. Subgroup and sensitivity analyses were also conducted.

**Result:**

Twelve studies met our inclusion criteria (*n* = 904). BTX-A injection showed greater sweat reduction compared to placebo by gravimetric measurement (116.12 [92.68–139.57]; *P *< 0.05). BTX-A injection showed comparable sweat reduction to other treatments by gravimetric measurement (26.14 [− 26.8, 79.07]; *P *= 0.333) and HDSS (− 0.85 [− 1.20, 0.50], *P *= 0.413). The pain score of BTX-A injection is comparable to other treatments (− 0.41 [− 1.11, 0.29], *P *= 0.456). BTX-A injection exhibited fewer side effects compared to other treatments (0.18 [0.07, 0.43], *P *< 0.05).

**Conclusion:**

In primary axillary hyperhidrosis, BTX-A injection significantly reduces sweat production compared with placebo, and there is no statistically significant difference compared with other treatments. BTX-A injection had less fewer side effects compared with other treatments.

**Level of Evidence I:**

This journal requires that authors assign a level of evidence to each article. For a full description of these Evidence-Based Medicine ratings, please refer to the Table of Contents or the online Instructions to Authors www.springer.com/00266.

**Supplementary Information:**

The online version contains supplementary material available at 10.1007/s00266-025-04909-6.

## Introduction

PAH is a focal, bilateral excessive sweating disorder that primarily affects the axillae. It is not associated with body thermal regulation and is usually triggered by emotional stress [1]. The prevalence of PAH is consistent across dermatology outpatient setting, regardless of geographical location, but varies with ethnicity, age, body mass index and sex [2]. The condition equally affects both men and women [3]. PAH significantly impact daily activities, work functions, and social interactions [4]. Patients with PAH often experience higher rates of depression and anxiety that occur compared to healthy individuals [5].

Antiperspirants are regarded as the first-line therapy for primary focal PAH and can provide significant benefit. However, there is no evidence that links the use of aluminum-containing antiperspirants to Alzheimer disease and no clear link exists between the use of aluminum-containing antiperspirants and breast cancer [6]. BTX-A injection has been shown to be effective in treating PAH by blocking the presynaptic release of acetylcholine [7]. Other treatment options for PAH include oral medication, microwave, ultrasound, laser, radiofrequency, local surgical procedures and sympathectomy [6]. Common qualitative methods for the evaluation include the Hyperhidrosis Disease Severity Scale (HDSS), gravimetry sweat production analysis, iodine starch testing and Hyperhidrosis Impact Questionnaire (HIQ) [8, 9].

Despite the absence of a relevant systematic review, this study aims to evaluate the effectiveness and safety of various treatments for PAH. We included 12 studies, divided into two subgroup analyses from randomized controlled trials with BTX-A and non-BTX-A therapies, to provide new evidence regarding the efficiency and safety of treatment of PAH treatment [10–21].

## Method

### Protocol and Guidance

This study was conducted in accordance with Preferred Reporting Items for Systematic Reviews and Meta-Analysis (PRISMA) guidelines. The protocol for this review was registered with PROSPERO (CRD42023454551) [22].

#### Inclusion Criteria

Studies were included in the meta-analysis when they met the following criteria: (1) The study was a randomized controlled trials (RCTs) that reported on the efficacy and safety of botulinum toxin A (BTX-A) injections in patients suffering from PAH. (2) The intervention time lasted for at least 2 weeks. (3) The article was published in the English language. (4) Eligible studies had to compare BTX-A injection with placebo or other treatment alternatives.

#### Exclusion Criteria

We excluded studies if the research type was not clearly defined, if valid outcome data could not be extracted from the article, if the full text could not be retrieved, or if the focus was on second axillary hyperhidrosis.

#### Outcome Measures

The primary outcome was gravimetric sweat reduction. Secondary outcomes included Hyperhidrosis Disease Severity Scale Scores (HDSS) and Visual Analog Scale (VAS) scores. Definitions of these outcomes are provided in eTable 1.

#### Search Strategy

In this meta-analysis and systematic review, we adhered to the PRISMA guidelines. A thorough and comprehensive literature search was conducted without language or date restrictions within databases including PubMed, Embase, Web of Science, and the Cochrane Library, covering publications up until May 2024. The search strategy is presented in eTable 2.

#### Study Selection

Two review authors (SJ and LPH) independently evaluated the titles and abstracts of all articles identified in the search. Only the studies that met the inclusion and exclusion criteria were eligible for inclusion, and then the full texts of the eligible studies were reviewed by the two authors independently. Disagreements between the two authors were resolved by another reviewer (T.Y).

#### Data Extraction and Quality Assessment

Two independent researchers (SJ and LPH) extracted data from the included trials using standardized data extraction tables. This approach ensured consistency and reduced the potential for error or bias in the data collection process. Both researchers have significant experience in evidence-based medicine and are well-versed in the nuances of data extraction for systematic reviews and meta-analyses.

In instances where a randomized controlled trial reported multiple outcome measures (e.g., 2 or 3), we summarize and include all of them; event outcomes in our analysis. This comprehensive approach provides a broader perspective on the efficacy and safety profiles of the interventions assessed.

When confronted with insufficient data in a particular study, we contacted the corresponding author to request the missing information. This proactive measure was taken to ensure that the meta-analysis was as comprehensive and accurate as possible, thereby enhancing the validity of our conclusions.

Any disagreement between two researchers during the data extraction process were resolved through consensus. This involved engaging in detailed discussions to understand each other's perspectives and using critical thinking to reach a mutually acceptable decision. This process helped to mitigate personal biases and ensured that the data extracted were both accurate and reliable.

### Statistical Analyses

We conducted statistical analyses using RevMan5.4, a software specifically designed for meta-analyses, which offers robust features for data synthesis and analysis. Additionally, to independently evaluate the quality of the included randomized controlled trials (RCTs), two reviewers utilized the Cochrane Collaboration’s risk of bias tool, ensuring a systemic and standardized assessment of study quality [23]. The meta-analysis was performed on a dataset derived from a total of 12 including studies. In cases where significant heterogeneity was present among the studies (*I*^2^ > 50%), we employed random-effects models for the meta-analysis.

#### Subgroup Analyses

Included studies were divided into 2 subgroups based on the different measurement received by the control group (placebo versus non-placebo). This method provided a more nuanced understanding of the intervention’s efficacy and safety in relation to the choice of control group.

#### Sensitivity Analyses

We conducted sensitivity analyses to test the robustness of the meta-analysis results by excluding trials with high or unknown risk of bias. We excluded trials with high risk of bias in one or more domains, unknown risk of bias with an unclear risk of bias in key domains where the information provided was insufficient to make a definitive assessment, as well as quasi randomized or cluster randomized trials.

After excluding the studies based on the aforementioned criteria, we recalculated the pooled effect estimates and confidence intervals for both the primary and secondary outcomes using the random-effects model. This allowed us to compare the results of the sensitivity analyses with those of the main analysis to determine if excluding studies with a high or unknown risk of bias significantly altered the conclusions.

## Results

### Eligible Studies and Study Characteristics

**Fig.1:**
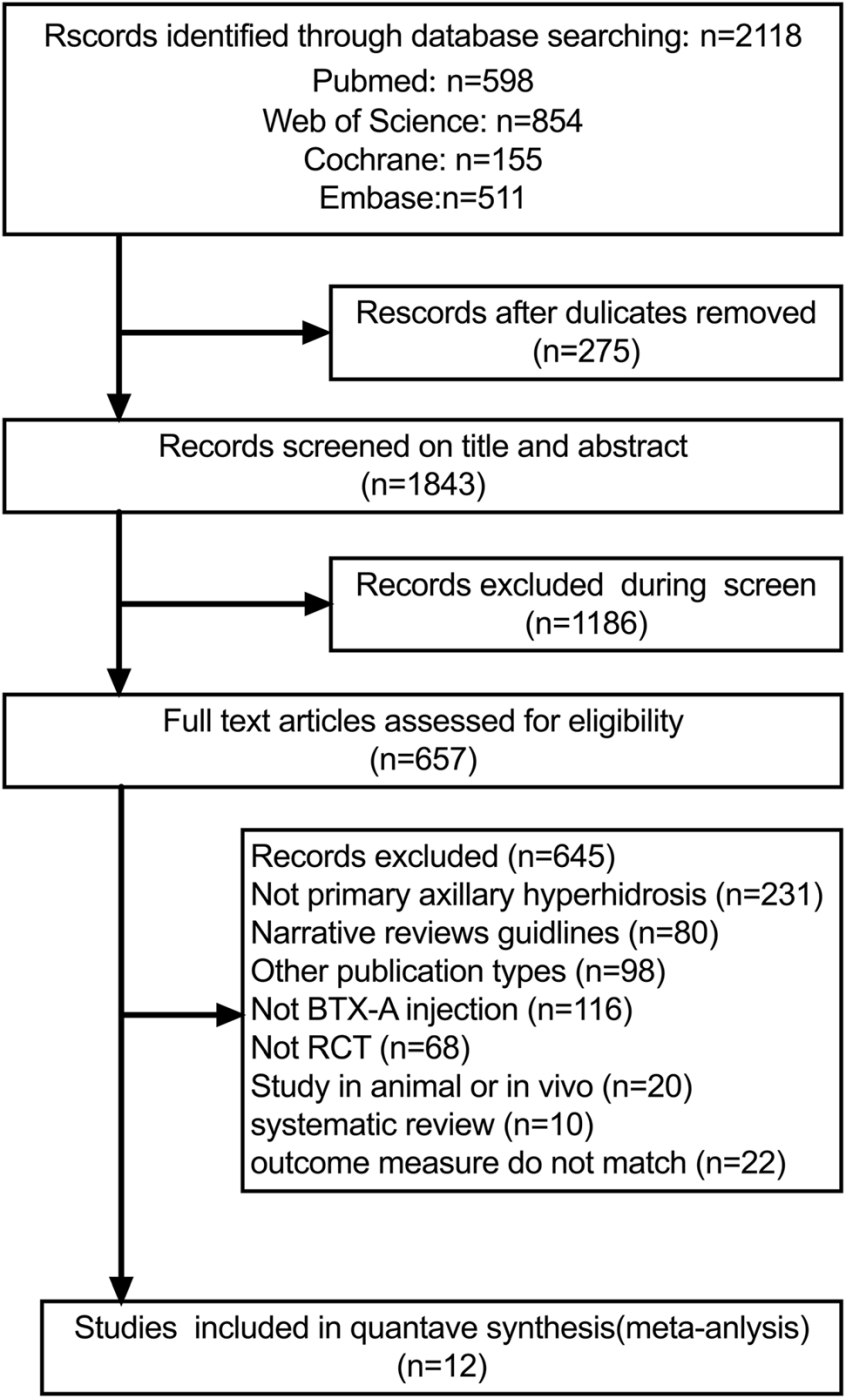
PRISMA flow diagram of the study selection process

The literature screening process is depicted in the PRISMA flowchart [24] (Fig. 1). The initial literature search identified a total of 2118 articles. After recognizing and eliminating 275 duplicate entries, we were left with 1843 unique studies. From these, 1186 articles were excluded based on title and abstract evaluation, and a further 657 articles were excluded after full-text screening against predefined exclusion criteria. Consequently, 12 studies were included in our meta-analysis for final evaluation [10–21].

A total of 904 patients were included across included studies, with 319 patients undergoing bilateral axillary (left and right) control. All subjects suffered from primary axillary hyperhidrosis and received BTX-A injections as treatment. The analyses compared the effectiveness of BTX-A with other treatment including microwave therapy, 20% aluminum chloride, iontophoresis, fractional microneedle radiofrequency and botulinum toxin B. Table 1 summarizes the details of the nine eligible studies, providing information on the number of patients, treatment types, and other relevant characteristics.

**Table 1:** Overview of study characteristics

Source	Country	Study design	Age BTX/Control	Male/Female BTX-A (Control)	BTX-A manufacturer/BTX-A dose per side	Manufacturer/control treatment	Follow up	Prolong duration
M Naumann 2001	Germany	RCT	Not mentioned	242 (total)	Allergan, Irvine/50U	placebo	1, 4, 16 week	At least 16 weeks
M. Naumann 2003	Germany	RCT	Not mentioned	203 (total)	Not mention/50u	placebo	16 months	30.6 (15.4–51.3) weeks
Marc Heckmann 2001	Germany	RCT (half-sided)	31.8 ± 11.0	76/69	Not mentioned/100u	placebo	14 weeks	Not mentioned
Paisal Rummaneethorn 2020	Thailand	RCT (half- sided)	36.8 ± 9.8	0/20	Not mentioned/50u	Not mentioned/Fractional microneedle radiofrequency	2, 14, 24 weeks	At least 24 weeks
Siri Archawawat 2023	Thailand	RCT (half- sided)	30.3 ± 3.2	6/6	Not mention/50u	Not mentioned/BTX-A25u	2, 4, 12 weeks	Not mentioned
Jee Soo An 2015	Korea	RCT (half- sided)	31.4 ± 7	11/13	Not mentioned/50u	Solstice Neuroscience/Botulinum toxin B	2, 12, 20 weeks	Not mentioned
Gabriela Lladó Grove 2024	Denmark	RCT (half- sided)	26 ± 16.8	10/20	Allergan 50-100u	Not mention/Microwave thermolysis	6, 12 months	Not mentioned
Katherine H 2008	America	RCT	29.0 ± 8.8/30.9 ± 7.2	5/20 (9/16)	Allergan/50	Person & Covey/Topical 20% aluminum chloride	2, 8, 12 weeks	Not mentioned
Diaa Aldin Sayed Ibrahim 2021	Egypt	RCT	37.9 ± 11.7/37.8 ± 12.12	8/12 (7/13)	Allergan/50u	Not mention/diode laser 980 nm	1, 6 months	Not mentioned
Sérgio Talarico-Filho 2007	Brazil	RCT (half- sided)	33.4 (19–56)	4/6	Allergan/50u	Ipsen Pharma BTX-A/150u	1 month	Not mentioned
Marc Heckmann 2005	Germany	RCT (half- sided)	35	19/18	Ipsen Pharma/100u	Ipsen Pharma/200u	2, 12, 36, 48 weeks	Not mentioned
Laleh Montaser-Kouhsari 2014	Iran	RCT (half- sided)	29.636 ± 6.021	5/6	Ipsen Pharma/250u	Ipsen Pharma/iontophoresis of BTX-A 250u	1 week, 1, 16 months	Not mentioned

### Quality Assessment

All studies included in the meta-analysis were assessed using Cochrane’s Collaboration’s tool. According to our inclusion criteria, all of these studies were randomized controlled trials (RCTs).. Allocation concealment was described in 83.33% of the included studies, indicating a majority of studies adequately concealed allocation to prevent selection bias. However, there are only 66.67% of the studies were double-blinded, suggesting that a third of the studies may have been susceptible to performance bias. No studies exhibited selective reporting, implying that all reported outcomes were those planned in the protocols, and there was no evidence of selective outcome reporting bias. Overall, the quality of the included studies was mixed. eFigures 1 and 2 demonstrate the results of the risk bias assessment.

#### Primary Outcome: Gravimetric Sweat Reduction

This meta-analysis includes 7 studies that uses gravimetric sweat reduction as the outcome measure. Upon conducting heterogeneity testing, we found *I*^2 ^= 90.20% > 50%, *P *< 0.05, which indicates strong heterogeneity among the included literature; therefore, we selected a random-effects model for our meta-analysis. The results for gravimetric sweat reduction were robust in sensitivity analyses (eFig.3). Funnel plot analysis showed no asymmetry (eFig 4), suggesting no evidence of publication bias.

**Fig. 2:**
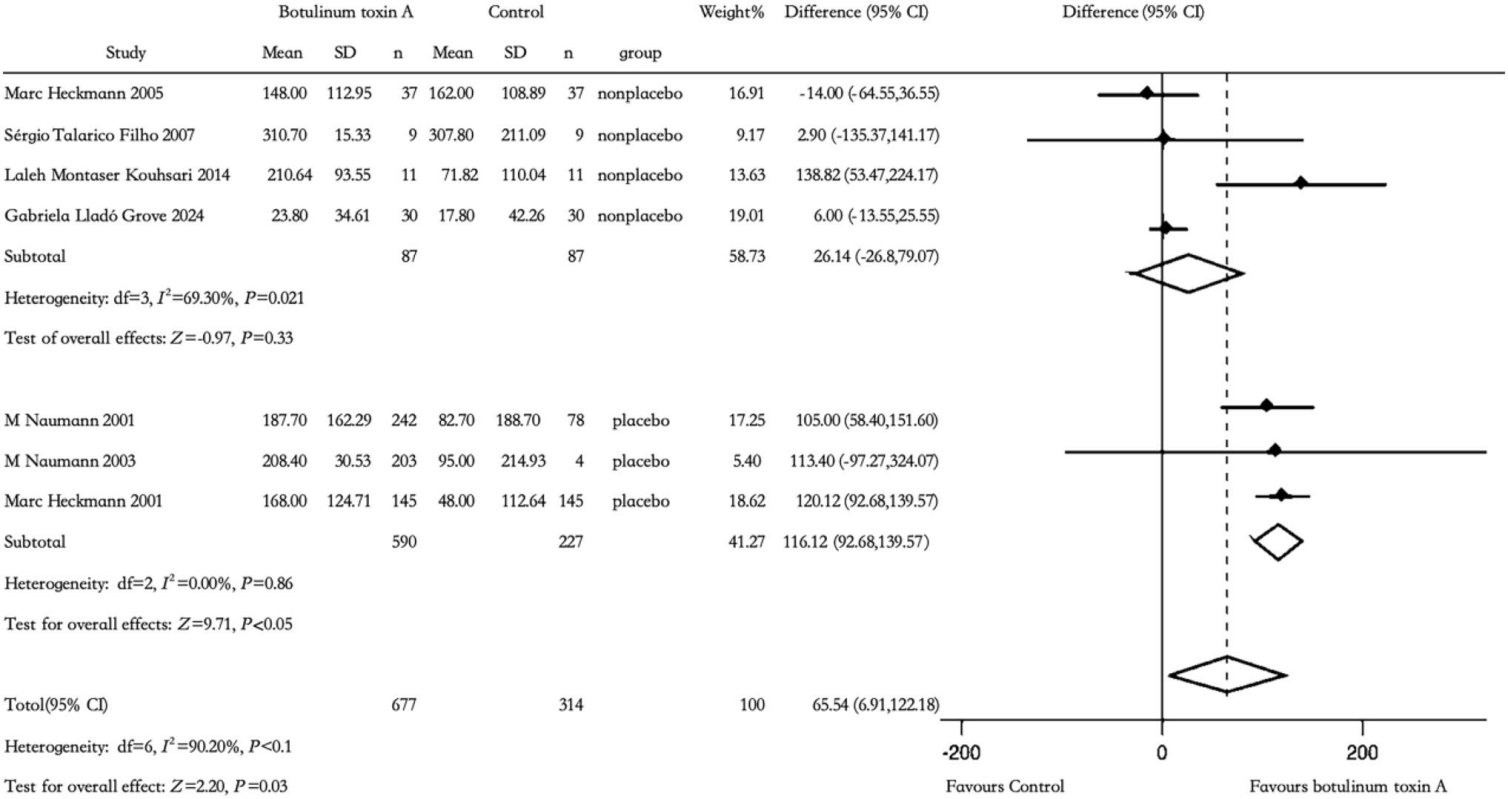
Comparison of BTX-A injection versus placebo and non-placebo: gravimetric sweat reduction after 2 weeks

The seven studies using gravimetric sweat reduction as outcome measurement were divided into 2 subgroups: placebo and non-placebo. The BTX-A group showed a significantly greater decrease than the placebo group at 116.12 mg/min after 2 weeks, with a notable difference in efficacy (Risk Difference (RD) 116.12, 95% confidence interval (CI) [92.68, 139.57], *Z* = 9.708, *P* < 0.05, *I*^2^ = 0.00%, 3 studies). There was no significant difference between BTX-A and non-placebo (one study versus difference doses and 1 study versus different manufacturers of BTX-A [14, 21], one study versus microwave thermolysis [12], one study versus iontophoresis of BTX-A [16],) in efficacy (RD 26.14, 95% CI [− 26.8, 79.07], *Z* = 0.968, *P* =0.33, *I*^2^ = 69.3%, 4) (Fig. 2).

#### Secondary Outcome: HDSS (Hydrodex Measurement of Axillary Sweat)

**Fig. 3:**
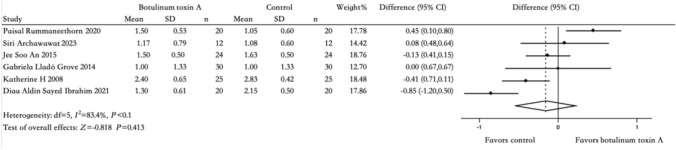
Comparison of BTX-A injection versus other treatments: HDSS after 2 weeks

Six studies measured the secondary outcome using HDSS. One study utilized both gravimetric sweat reduction and HDSS as outcome measures. After conducting heterogeneity testing, *I*^2^=83.4% > 50%, *P *< 0.1, indicating strong heterogeneity among included literature; therefore, we selected random-effects model for our meta-analysis (Fig 3). The meta-analysis results for HDSS were robust in sensitivity analyses (eFig. 5). Funnel plot analysis showed no asymmetry (eFig. 6), suggesting no evidence of publication bias.

Among the six included studies, one compared fractional microneedle radiofrequency [19], another compared botulinum toxin B [10], the third studied microwave thermolysis [12], the fourth evaluated topical 20% aluminum chloride [11], and the fifth examined diode laser treatment between BTX-A and other interventions using HDSS as an outcome measurement. The RD was − 0.85, with a 95% CI pf [− 1.20, 0.50], *Z* = 0.818, *P *= 0.413, *I*^2^ = 83.4%, 6 studies.

### Side Effects

**Fig. 4:**
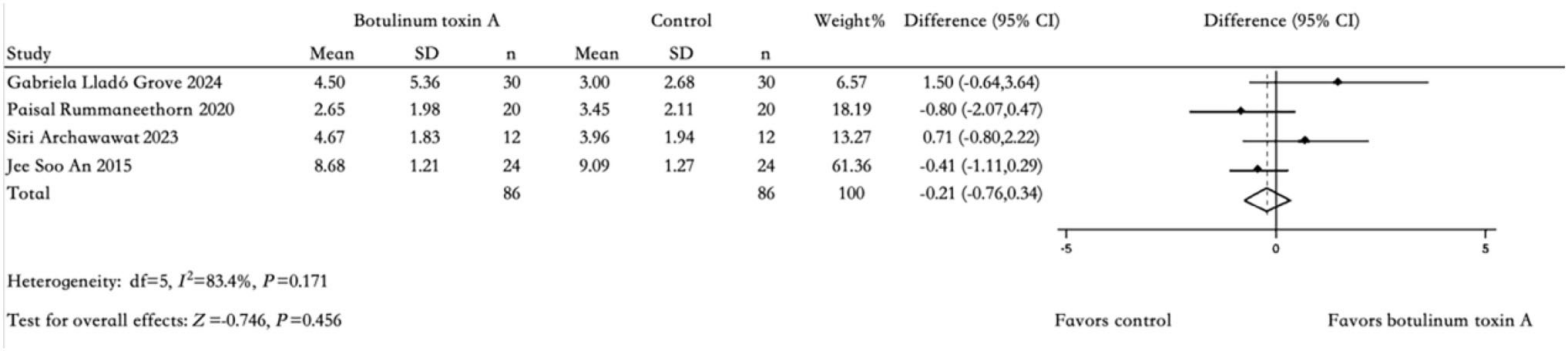
Comparison of BTX-A injection versus other treatment: pain score after 2 weeks

**Fig. 5:**
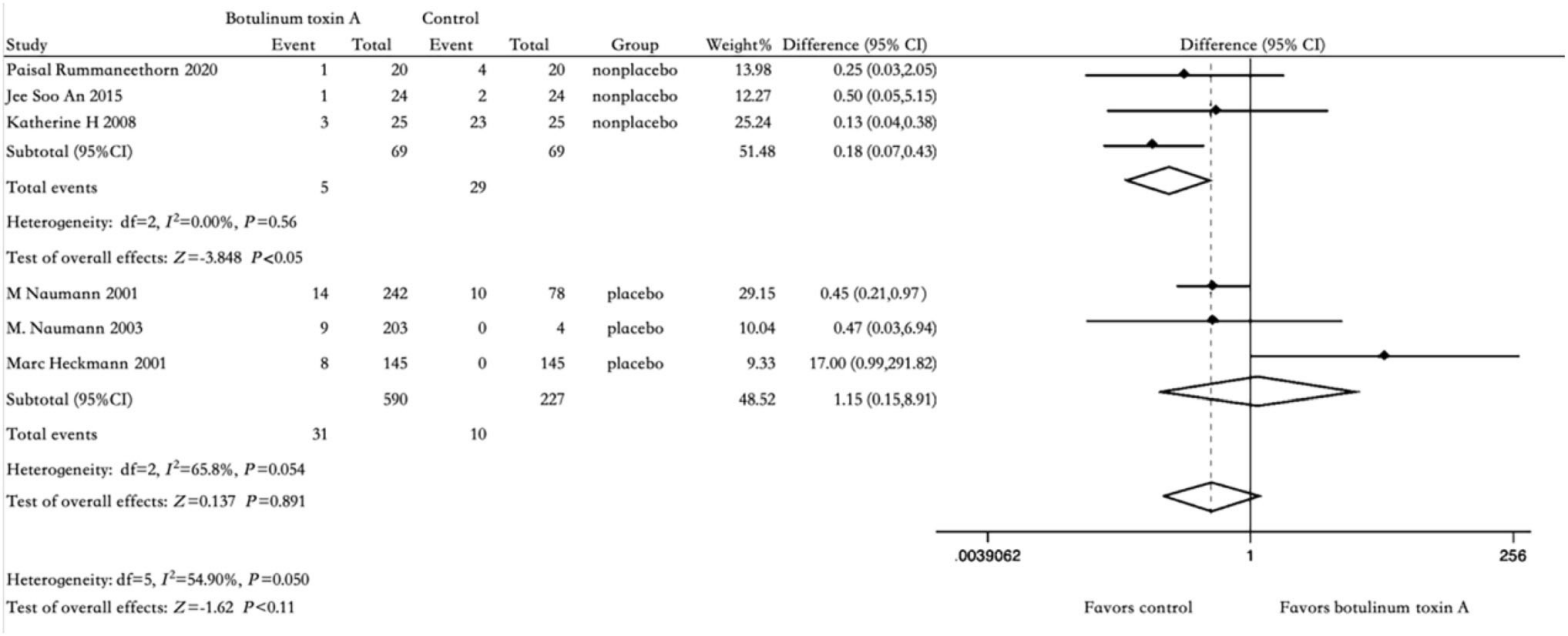
Comparison of BTX-A injection versus placebo and non-placebo: side effect after 2 weeks

Four studies measured pain score using the Visual Analog Scale (VAS) [10, 12, 19, 20]. The pain score in the BTX-A group was comparable to other treatments, showing no statistical significance (RD − 0.21, 95% CI [− 0.76, 0.34], *Z *= 0.746, *P *= 0.456, *I*^2 ^= 83.4%, 6 studies) (Fig 4). Six studies reported specific side effects [10, 11, 13, 17–19]. We divided into two subgroups (placebo and non-placebo). Compared with placebo, the adverse event incidence of BTX-A injection was comparable, showing no statistically significant difference (RD 1.15, 95% CI [0.15, 8.91], *Z *= 0.137, *P *= 0.891, *I*^2 ^= 65.8%, 3 studies) (Fig 5). Compared with non-placebo treatments, the adverse incidence of BTX-A injection was significantly lower than non-placebo group, demonstrating a statistical difference (RD 0.18, 95% CI [0.07, 0.43], *Z* = 3.848, *P *< 0. 05, *I*^2^ 0.00%, 3studies) (Fig 5).

## Discussion

Primary axillary hyperhidrosis (PAH) is a dermatologic disorder characterized by sweat production exceeding thermoregulatory needs [9]. The etiology of PAH may stem from a complex autonomic nervous system dysfunction, resulting in neurogenic overactivity of otherwise normal eccrine sweat glands [9]. PAH significantly reduces patients' quality of life and even lead to anxiety [25].

The commonly used treatment options for PAH included: topical antiperspirants, BTX-A injections, oral medications, microwave, ultrasound, laser, radiofrequency, local surgical procedures, sympathectomy [6]. However, the final treatment plan depends on various factors such as the severity, safety, side effects, extent of irreversibility, cost, treatment availability, and the provider’s expertise and experience.

Our meta-analysis results show promising implications for patients seeking medical help for the treatment of PAH. Based on mostly moderate quality evidence from three RCTs, the average sweat secretion decreased by 116.12 mg after 2 weeks of BTX-A injection, as measured by gravimetric sweat reduction test (*P *< 0.05). Other treatments, including microwave thermolysis, iontophoresis of BTX-A, botulinum toxin B, topical 20% aluminum chloride and diode laser, were also effective in treating PAH, whether evaluated by gravimetric sweat reduction test or HDSS (*P *> 0.05).

Our meta-analysis suggests the BTX-A injection is as effective as other treatments, including microwave thermolysis, iontophoresis of BTX-A, botulinum toxin B, topical 20% aluminum chloride, diode laser 980 nm and different dose of BTX-A, in treating PAH. However, the shortcomings of this study lie in the lack of relevant reports on the maintenance time of BTX-A injection and the limited number of studies on other treatment options. Notably, different treatments yield varied outcomes. For example, one randomized controlled trial compared BTX-A with microwave thermolysis and found no significant difference in efficacy between the two treatments [12]. Another trial compared BTX-A injection with iontophoresis of BTX-A, and found BTX-A injection to be more effective [16]. A trial comparing BTX-A injection with botulinum toxin B (BTX-B) showed both BTX-A and BTX-B showed comparable anhidrotic effects at a conversion ratio of 1:30 through 20 weeks [10]. Furthermore, BTX-A found to be more effective and provided greater patient satisfaction than topical 20% aluminum chloride at week 4 [11], and diode laser 980 nm was found to be more effective than BTX-A injection [15]. Different doses (50u vs. 25u) and different manufactures (Botox vs. Dysport) were found to have the same therapeutic effect [14, 14, 21]. Overall, our study suggests that BTX-A injection is as effective as the non-placebo treatment, but more research is needed.

Our meta-analysis indicates that the incidence of adverse reactions with BTX-A injection is comparable to that in the placebo group and lower compare to other treatment options. The main adverse reaction of BTX-A is pain, with the pain score of BTX-A injection is equivalent to other treatment options (*P *> 0.05). Other adverse reactions of BTX-A injection included infection and common cold (*n *= 14), non-axillary hyperhidrosis (*n *= 12), headache (*n *= 4), itching (*n *= 3), shoulder girdle muscle soreness (*n* = 2), axillary itching (*n *= 1) and underarm purpura (*n *= 1). However, the non-placebo group treatment had more adverse reactions, including burning, itching and redness (*n *= 23), non-axillary hyperhidrosis (*n* = 2), infection and common cold (*n *= 10), and underarm purpura (*n *= 1). No studies reported BTX-A poisoning or allergic shock. Three studies reported the maintenance time of BTX-A injection: at least 14 weeks, at least 16 weeks, and 30.6 (15.4–51.3) weeks.

## Limitations of the Study

The present meta-analysis, while synthesizing the current evidence on the treatment of PAH with BTX-A, encounters several limitations that must be considered when interpreting the findings. Firstly, there is a scarcity of studies reporting the long-term maintenance effects of BTX-A injections. Understanding the duration of effectiveness is crucial for both clinicians and patients in deciding the frequency and necessity of repeated interventions.

Additionally, the dosage variation of BTX-A and the potential differences in efficacy between different manufacturers (e.g., Botox vs. Dysport) have not been thoroughly investigated. The conversion ratio of BTX-A to BTX-B and its implications in clinical practice likewise require more robust evidence.

Lastly, the adverse reaction profile of BTX-A, although generally favorable compared to placebo, needs to be assessed in larger cohorts to determine its true incidence and severity. This is particularly important for patients and practitioners when balancing the risk-benefit ratio of treatments.

In conclusion, while this meta-analysis provides insights into the treatment of PAH with BTX-A, future research should focus on large-scale RCTs with consistent outcome measures and longer follow-up periods to validate these findings and guide clinical decision-making.

## Conclusion

Our data suggest that BTX-A effectively yields superior results for patients with PAH in terms of subjective and quantitative analysis compared to placebo. Besides, our results hint the BTX-A injection is effective as other treatments (e.g., microwave thermolysis, iontophoresis of BTX-A, botulinum toxin B, topical 20% aluminum chloride, diode laser 980 nm and different dose of BTX-A). The adverse reactions of BTX-A injection are comparable to placebo, and compared to other treatment options, BTX-A injection has fewer adverse reactions.

In synthesizing the current evidence, our data suggest that BTX-A offers superior outcomes for patients suffering from PAH. Both subjective and quantitative analyses reveal the efficacy of BTX-A compared to placebo, affirming its clinical relevance and utility in the management of this condition. Furthermore, when compared with alternative treatments such as microwave thermolysis, iontophoresis of BTX-A, botulinum toxin B, topical 20% aluminum chloride, and diode laser 980 nm, BTX-A injection demonstrates comparable effectiveness. This equivalence in therapeutic outcomes underscores the versatility and adaptability of BTX-A in treating PAH.

It is reassuring to note that the adverse reaction profile of BTX-A injections closely mirrors that of the placebo. Notably, when compared to other treatment options, BTX-A injections exhibit fewer adverse reactions, further solidifying its position as a well-tolerated intervention.

In conclusion, BTX-A represents a valuable treatment option against PAH, offering efficacy and tolerability that is on par with, if not superior to, existing treatments. However, the limitations of the current evidence base necessitate cautious interpretation and highlight the need for larger, rigorous RCTs to corroborate these findings, as well as refine our understanding of the risks and benefits associated with BTX-A in PAH management.

## Supplementary Information

Below is the link to the electronic supplementary material.

Supplementary file1 (PDF 75 KB)

Supplementary file2 (PDF 85 KB)

Supplementary file3 (JPG 483 KB)

Supplementary file4 (JPG 1209 KB)

Supplementary file5 (JPG 171 KB)

Supplementary file6 (JPG 535 KB)

Supplementary file7 (JPG 387 KB)

Supplementary file8 (JPG 457 KB)
