# Supplementary material for: Efficacy and Safety of Botulinum Toxin Type A in Primary Axillary Hyperhidrosis: A Meta-analysis and Systematic Review
Source: Aesthetic Plast Surg. 2025 Jun 11;49(17):4932–40. doi: 10.1007/s00266-025-04909-6 (PMC12500766; doi:10.1007/s00266-025-04909-6)
Supplement: Supplementary file 1 — Supplementary file1 (PDF 75 KB) [file 266_2025_4909_MOESM1_ESM.pdf]

eable 1: Definitions of outcomes

**Outcomes**	**Definitions**
Gravimetric sweat reduction	The weight of sweat produced per minute before treatment minus the weight of sweat produced per minute after treatment.
Disease Severity Scale Scores (HDSS)	Tolerability of sweating and its effect on the patient's life on a 4-point scale：<br>Score 1: underarm seating never noticeable and never interferes with daily activities;<br>Score 2: underarm sweating is tolerable but sometimes interferes with daily activities;<br>Score 3: underarm sweating is barely tolerable and frequently interferes with daily activities;<br>Score 4: underarm seating intolerable and always interferes with daily activities.
VAS	visual analog scale score
