# Supplementary material for: Efficacy and Safety of Botulinum Toxin Type A in Primary Axillary Hyperhidrosis: A Meta-analysis and Systematic Review
Source: Aesthetic Plast Surg. 2025 Jun 11;49(17):4932–40. doi: 10.1007/s00266-025-04909-6 (PMC12500766; doi:10.1007/s00266-025-04909-6)
Supplement: Supplementary file 2 — Supplementary file2 (PDF 85 KB) [file 266_2025_4909_MOESM2_ESM.pdf]

eable 2: Search strategy

**PubMed**		
1	("Botulinum Toxins, Type A "[MeSH]) OR (Clostridium botulinum A Toxin) OR (Botulinum Toxin A) OR (Toxin A, Botulinum) OR (Botulinum Neurotoxin A) OR (Neurotoxin A, Botulinum) OR (Botulinum A Toxin) OR (Toxin, Botulinum A) OR (Botulinum Toxin Type A) OR (Botulinum Neurotoxin Type A) OR (Clostridium Botulinum Toxin Type A) OR (Meditoxin) OR (Botox) OR (Neuronox) OR (Oculinum) OR (Vistabex)) OR (OnabotulinumtoxinA) OR (Onabotulinumtoxin A) OR (Vistabel)	15176
2	("Hyperhidrosis"[MeSH]) OR (axillary hyperhidrosis) OR (primary axillary hyperhidrosis) OR (Underarm Sweating)	4848

3	Randomized controlled trial	826722
4	(1 and 2) and 3	598
**Web of Sscience**		
1	TS= (Hyperhidrosis) OR (axillary hyperhidrosis) OR (primary axillary hyperhidrosis)	4178
2	TS= (Botulinum Toxins, Type A) OR (Clostridium botulinum A Toxin) OR (Botulinum Toxin A) OR (Toxin A, Botulinum OR Botulinum Neurotoxin A) OR (Neurotoxin A, Botulinum) OR B(otulinum A Toxin) OR (Toxin, Botulinum A) OR (Botulinum Toxin Type A) OR (Botulinum Neurotoxin Type A OR Clostridium Botulinum Toxin Type A) OR (Meditoxin) OR (Botox OR Neuronox) OR (Oculinum) OR (Vistabex) OR (OnabotulinumtoxinA) OR OnabotulinumtoxinA OR Vistabe)	28694

3	1 and 2	854
**Cochrane**		
1	(Hyperhidrosis) OR (axillary hyperhidrosis) OR (primary axillary hyperhidrosis): ab,ti,kw	1136
2	(Botulinum toxins, type A) OR (Clostridium botulinum A toxin) OR (Botulinum toxin A) OR (toxin A, botulinum) OR (Botulinum neurotoxin A) OR (Neurotoxin A, botulinum) OR (Botulinum A toxin) OR (Toxin, botulinum A) OR (Botulinum toxin type A) OR (Botulinum neurotoxin type A) OR (Clostridium botulinum toxin type A) OR (Meditoxin) OR (Botox) OR (Neuronox) OR (Oculinum) OR (Vistabex OR OnabotulinumtoxinA) OR (Onabotulinumtoxin A) OR Vistabe):ab,ti,kw	9480

3	1 and 2	155
**Embase**		
1	("Hyperhidrosis": ab,ti) OR (“axillary hyperhidrosis":ab,ti) OR (“primary axillary hyperhidrosis": ab,ti)	1136
2	('Botulinum toxins, Type A": ab,ti) OR ("Clostridium botulinum A Toxin":ab,ti) OR (“Botulinum Toxin A": ab,ti) OR "Toxin A, Botulinum": ab,ti) OR "(Botulinum Neurotoxin A":ab,ti) OR ("Neurotoxin A, Botulinum":ab,ti) OR (“Clostridium botulinum A Toxin": ab,ti) OR ("Toxin, Botulinum A":ab,ti) OR ("Botulinum Toxin Type A":ab,ti) OR (“Botulinum Neurotoxin Type A": ab,ti) OR ("Clostridium Botulinum Toxin Type A":ab,ti) OR ("Meditoxin": ab,ti) OR ("Botox":ab,ti) OR ("Neuronox":ab,ti) OR ("Oculinum":ab,ti) OR ("Vistabex":ab,ti) OR ("Vistabex": ab,ti) OR	9480

	("OnabotulinumtoxinA":ab,ti) OR<br>(“Onabotulinumtoxin A":ab,ti) OR<br>("Vistabel":ab,ti)	
3	1 and 2	511
