# Supplementary figures and images for: Efficacy and Safety of Botulinum Toxin Type A in Primary Axillary Hyperhidrosis: A Meta-analysis and Systematic Review
Source: Aesthetic Plast Surg. 2025 Jun 11;49(17):4932–40. doi: 10.1007/s00266-025-04909-6 (PMC12500766; doi:10.1007/s00266-025-04909-6)

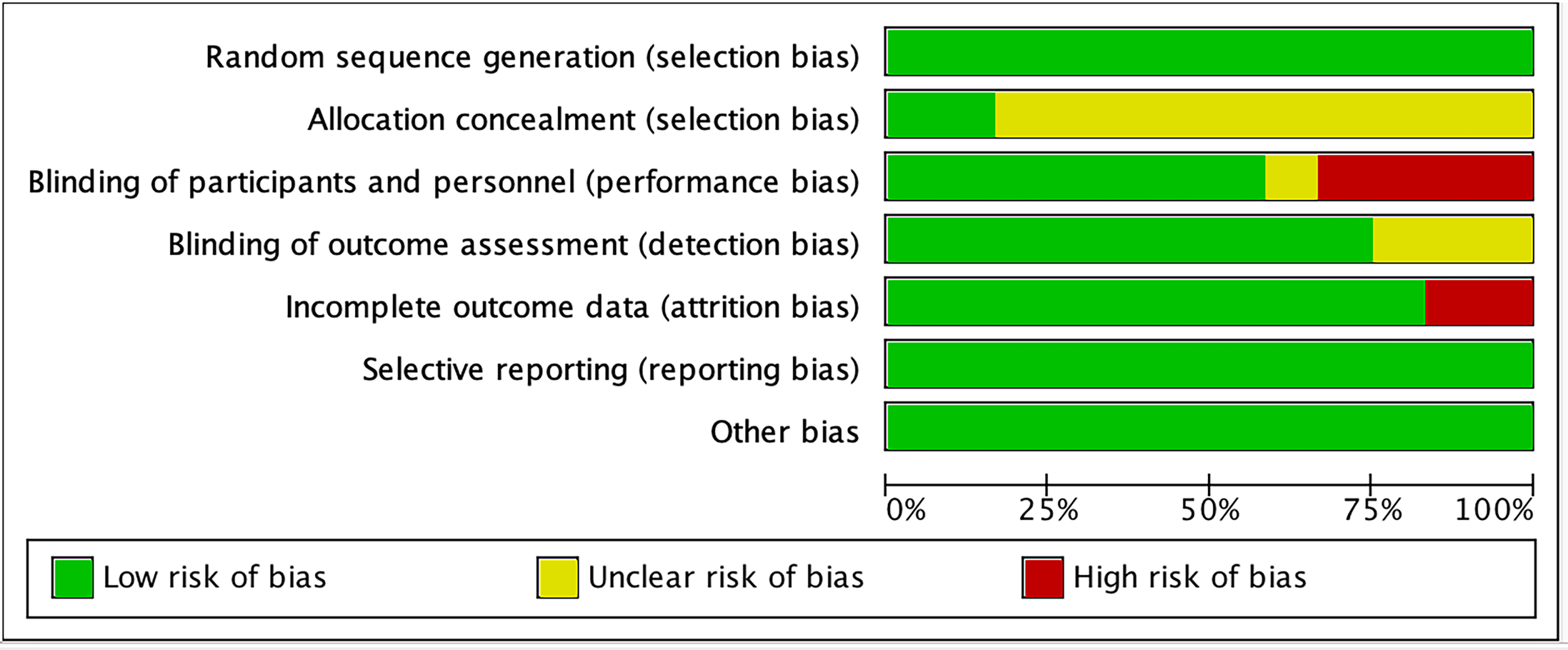

Supplement: Supplementary file 3 — Supplementary file3 (JPG 483 KB) [file 266_2025_4909_MOESM3_ESM.jpg]

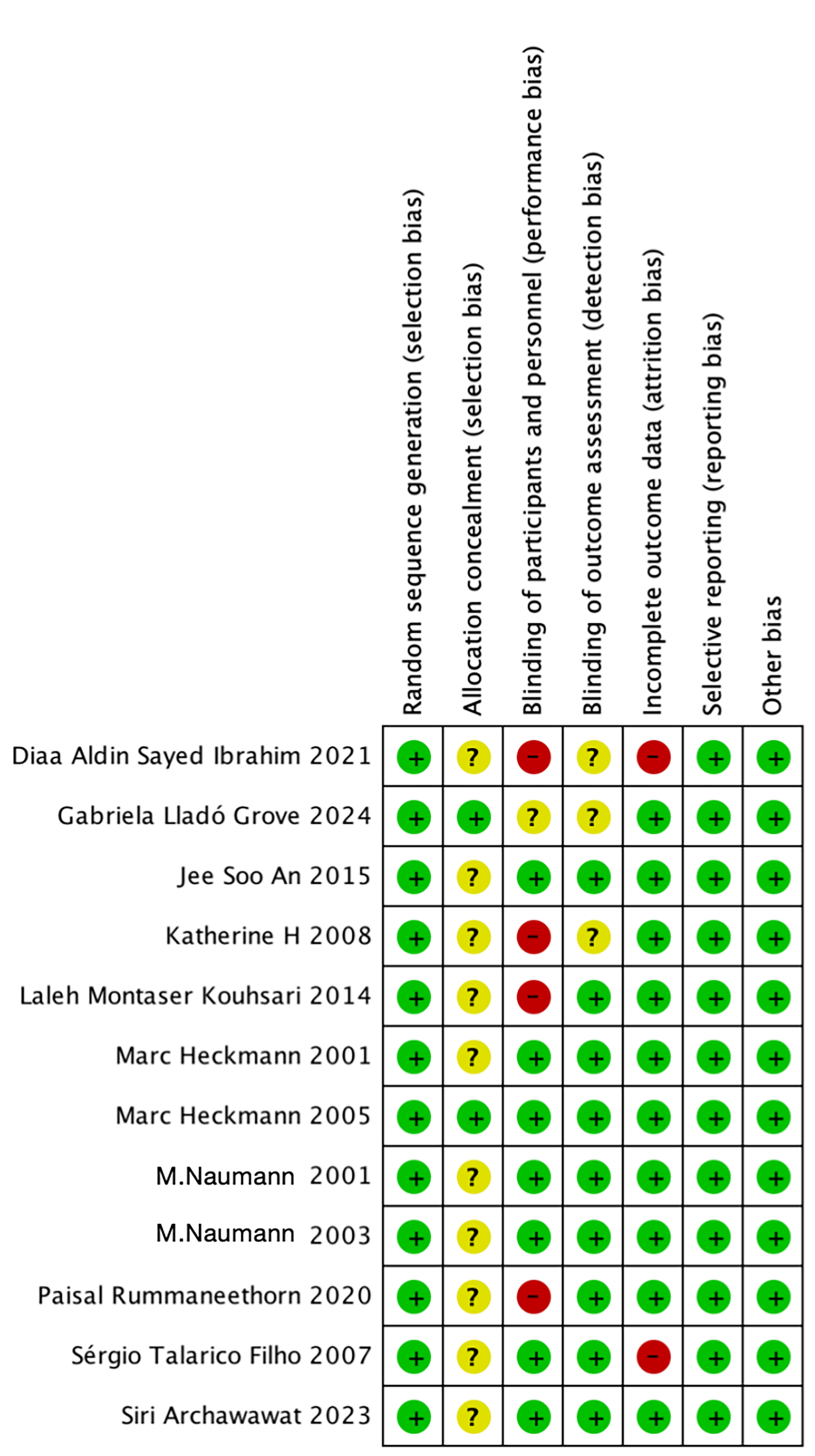

Supplement: Supplementary file 4 — Supplementary file4 (JPG 1209 KB) [file 266_2025_4909_MOESM4_ESM.jpg]

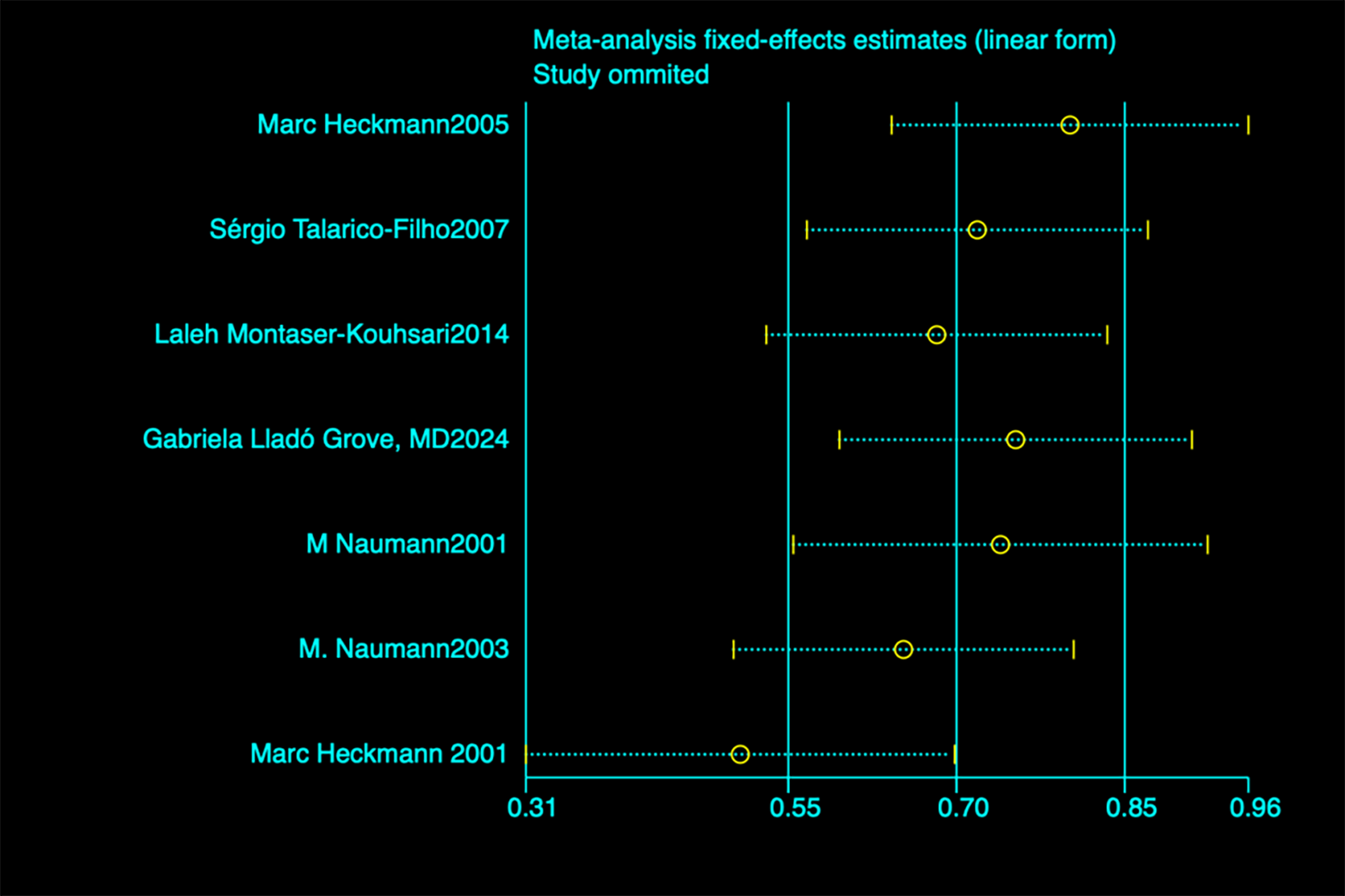

Supplement: Supplementary file 5 — Supplementary file5 (JPG 171 KB) [file 266_2025_4909_MOESM5_ESM.jpg]

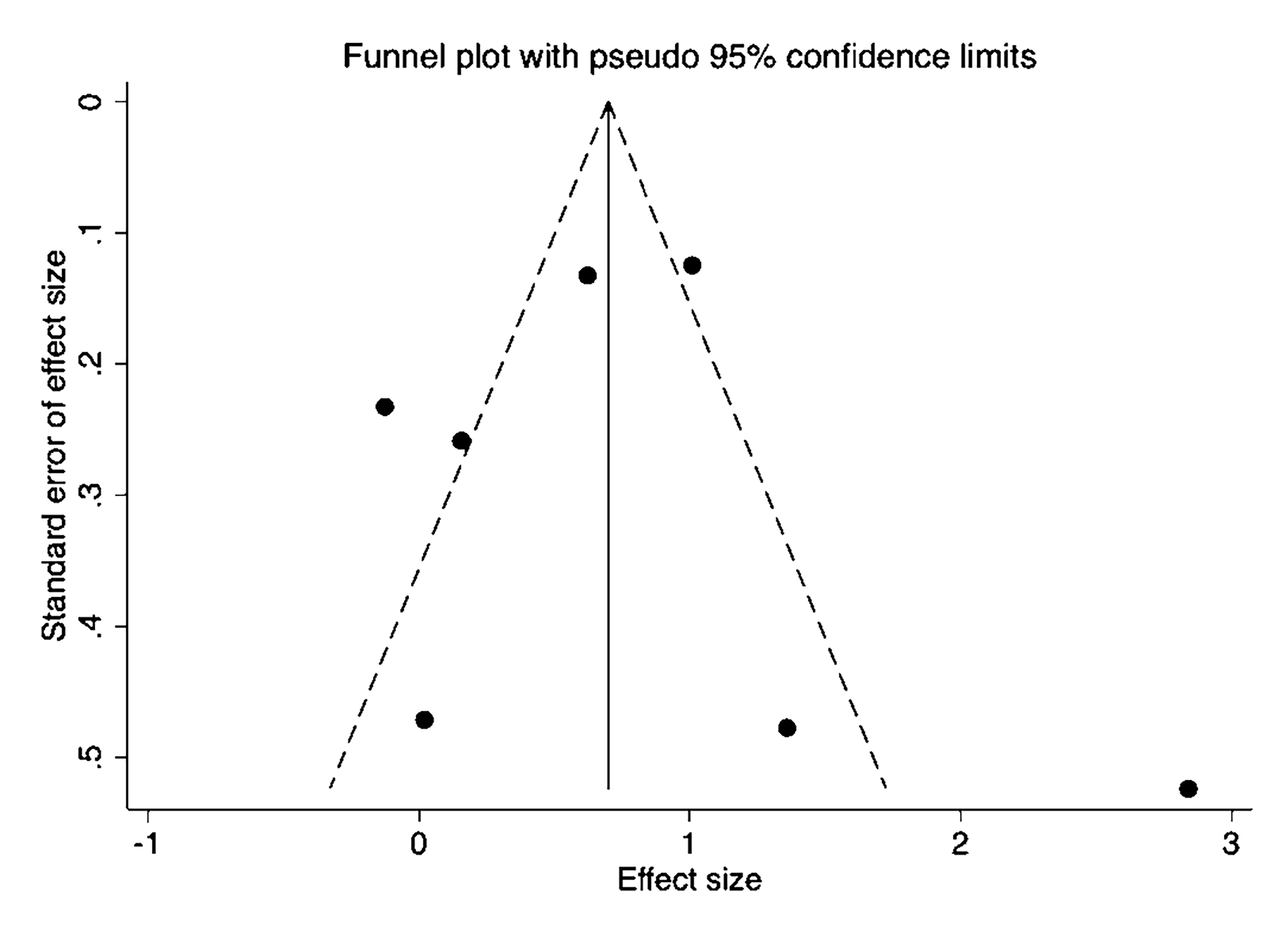

Supplement: Supplementary file 6 — Supplementary file6 (JPG 535 KB) [file 266_2025_4909_MOESM6_ESM.jpg]

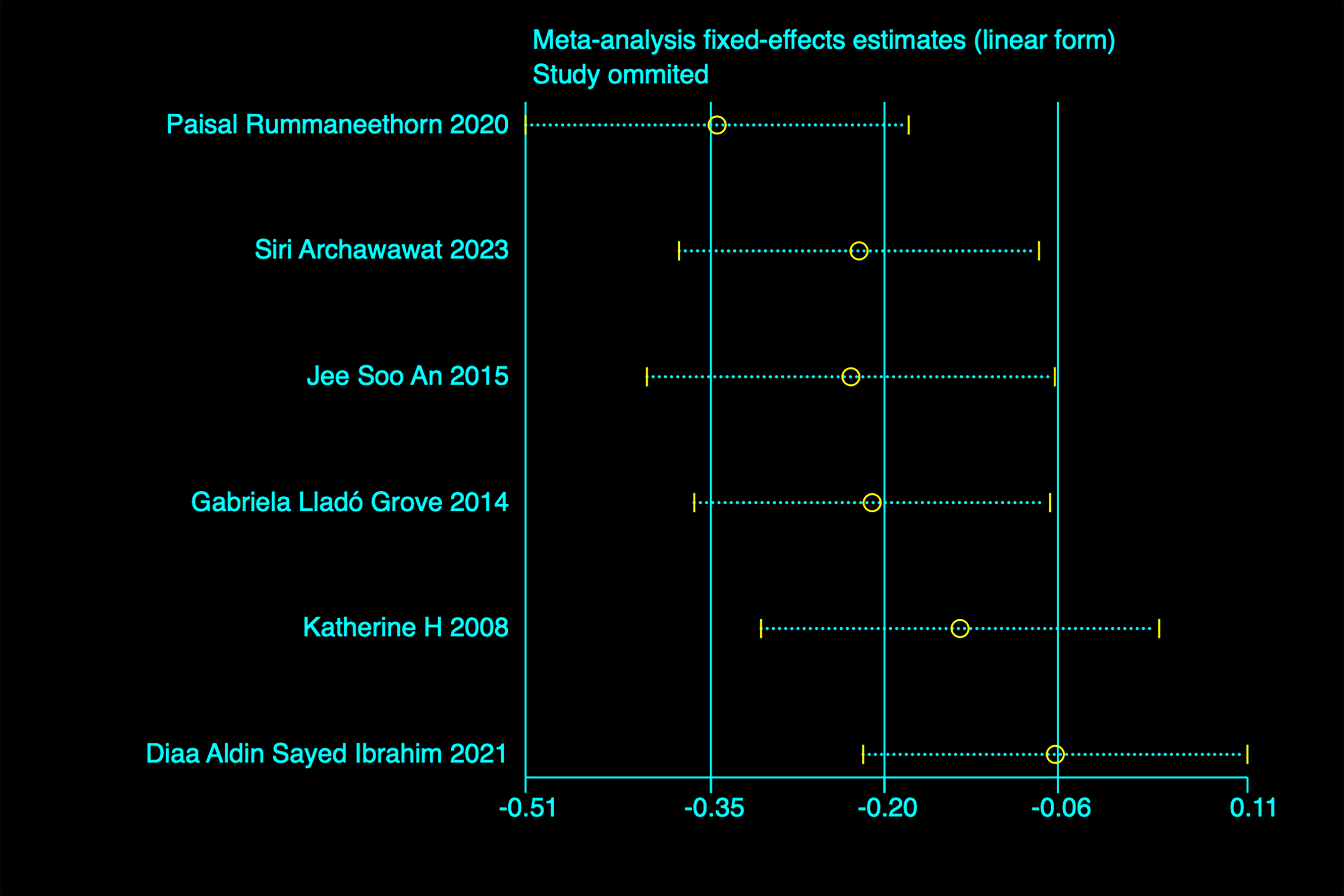

Supplement: Supplementary file 7 — Supplementary file7 (JPG 387 KB) [file 266_2025_4909_MOESM7_ESM.jpg]

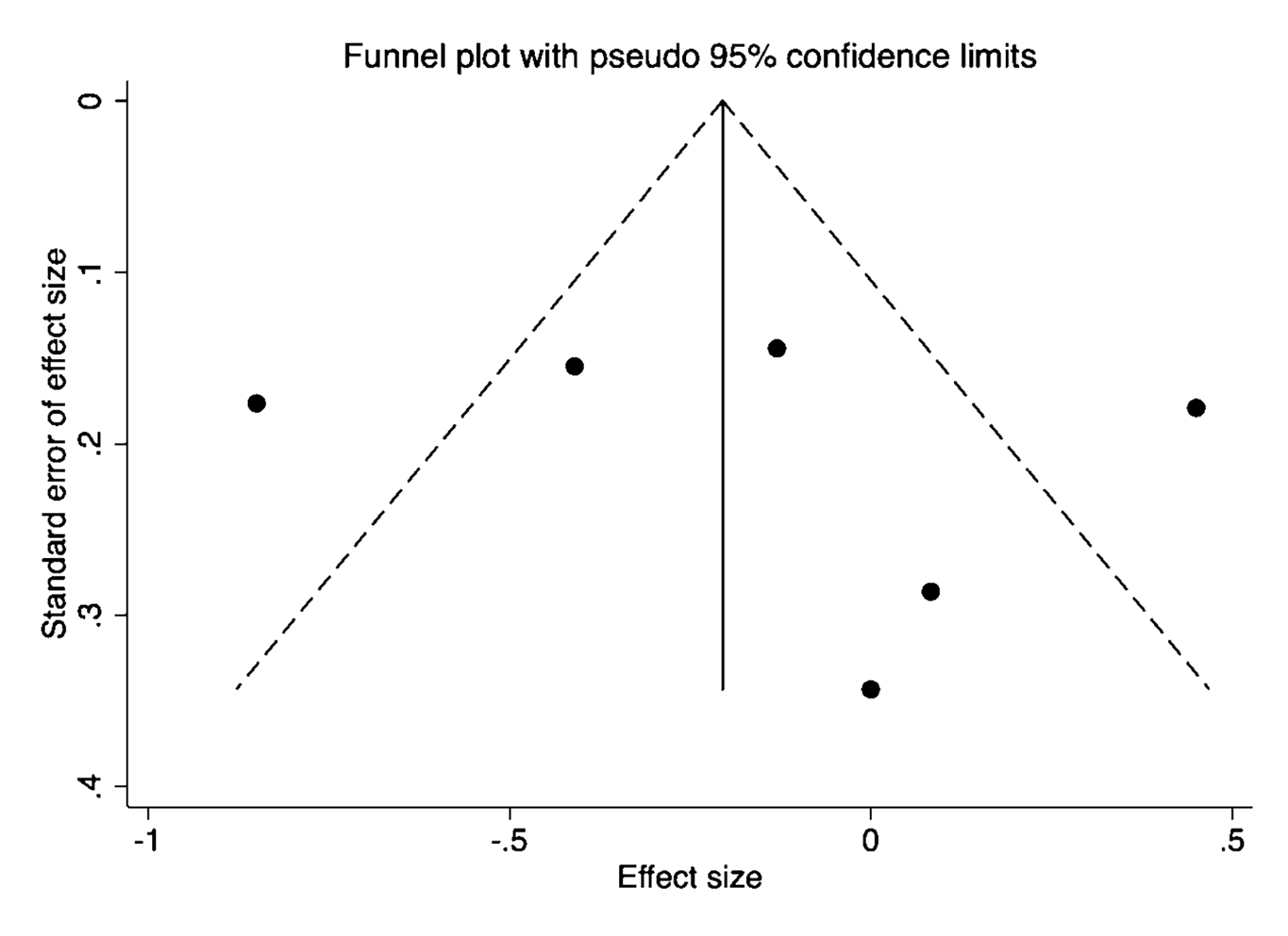

Supplement: Supplementary file 8 — Supplementary file8 (JPG 457 KB) [file 266_2025_4909_MOESM8_ESM.jpg]
